# Ablation of the *Id2* Gene Results in Altered Circadian Feeding Behavior, and Sex-Specific Enhancement of Insulin Sensitivity and Elevated Glucose Uptake in Skeletal Muscle and Brown Adipose Tissue

**DOI:** 10.1371/journal.pone.0073064

**Published:** 2013-09-02

**Authors:** Deepa Mathew, Peng Zhou, Cameron M. Pywell, Daan R. van der Veen, Jinping Shao, Yang Xi, Nicolle A. Bonar, Alyssa D. Hummel, Sarah Chapman, W. Matthew Leevy, Giles E. Duffield

**Affiliations:** 1 Department of Biological Sciences, University of Notre Dame, Notre Dame, Indiana, United States of America; 2 Notre Dame Integrated Imaging Facility, University of Notre Dame, Notre Dame, Indiana, United States of America; 3 Eck Institute for Global Health, University of Notre Dame, Notre Dame, Indiana, United States of America; Pennsylvania State University, United States of America

## Abstract

Inhibitor of DNA binding 2 (ID2) is a helix-loop-helix transcriptional repressor rhythmically expressed in many adult tissues. Our earlier studies have demonstrated a role for ID2 in the input pathway, core clock function and output pathways of the mouse circadian system. We have also reported that *Id2* null (*Id2*−/−) mice are lean with low gonadal white adipose tissue deposits and lower lipid content in the liver. These results coincided with altered or disrupted circadian expression profiles of liver genes including those involved in lipid metabolism. In the present phenotypic study we intended to decipher, on a sex-specific basis, the role of ID2 in glucose metabolism and in the circadian regulation of activity, important components of energy balance. We find that *Id2*−/− mice exhibited altered daily and circadian rhythms of feeding and locomotor activity; activity profiles extended further into the late night/dark phase of the 24-hr cycle, despite mice showing reduced total locomotor activity. Also, male *Id2−/−* mice consumed a greater amount of food relative to body mass, and displayed less weight gain. *Id2−/−* females had smaller adipocytes, suggesting sexual-dimorphic programing of adipogenesis. We observed increased glucose tolerance and insulin sensitivity in male *Id2−/−* mice, which was exacerbated in older animals. FDG-PET analysis revealed increased glucose uptake by skeletal muscle and brown adipose tissue of male *Id2*−/− mice, suggesting increased glucose metabolism and thermogenesis in these tissues. Reductions in intramuscular triacylglycerol and diacylglycerol were detected in male *Id2*−/− mice, highlighting its possible mechanistic role in enhanced insulin sensitivity in these mice. Our findings indicate a role for ID2 as a regulator of glucose and lipid metabolism, and in the circadian control of feeding/locomotor behavior; and contribute to the understanding of the development of obesity and diabetes, particularly in shift work personnel among whom incidence of such metabolic disorders is elevated.

## Introduction

Inhibitor of DNA binding 2 (ID2) is a dominant negative regulator of basic helix-loop-helix (bHLH) transcription factors, which acts by heterodimerizing with them and inhibiting their ability to bind to E-box elements within target gene promoters [1], [2]. A functional role of ID2 has been demonstrated in cell lineage determination, cell cycle progression, adaptive immunity and tumorigenesis [1], [2]. *Id2* is expressed in a many adult tissues and shows circadian rhythmicity in its RNA and protein expression [3], [4].

Circadian rhythms are cyclical occurrences of physiological and/or behavioral events with a periodicity of ∼24 hrs, exhibited by all eukaryotic organisms [5]. This intrinsic time keeping mechanism is believed to have evolved as an adaptive response to the daily changes in the surrounding environment such as light and food availability. This enables organisms to anticipate such changes and coordinate, or temporally partition, different biological processes for optimal utilization of resources. In mammals the master circadian oscillator resides in the hypothalamic suprachiasmatic nuclei (SCN). The SCN synchronizes and phase resets the cell autonomous clock mechanisms in the peripheral tissues through humoral signals, autonomic innervation, body temperature and feeding related cues [6]. At the molecular level, the clock is encoded by transcriptional-translational autoregulatory feedback loops of core clock proteins. The positive loop is composed of bHLH-PAS transcriptional activators CLOCK (or NPAS2) and BMAL1. They heterodimerize and transactivate a proportion of the downstream clock controlled genes (CCGs) responsible for clock output, as well as transactivate the transcriptional repressor genes *period* and *cryptochrome*, that constitute the negative loop [5].

Recent evidence reveals that circadian rhythms and metabolism are tightly intertwined physiological processes. The circadian clock regulates glucose homeostasis at the levels of both SCN and peripheral clocks. Daily rhythms are observed in blood glucose levels, glucose tolerance, insulin sensitivity and glucose uptake by the brain, skeletal muscle and adipose tissue [7]–[9]. Moreover, disturbances in the circadian rhythmicity and sleep-wake cycle in humans, such as in shift-work personnel, are associated with diabetes, metabolic syndrome, hypoleptinemia, increased appetite and obesity [7]. Furthermore genetic disruption of several canonical clock genes in animal models results in diverse and profound metabolic disturbances, demonstrating the involvement of clock genes in glucose homeostasis [7].

Our previous studies have demonstrated that ID2 has roles in core clock function by interacting with CLOCK and BMAL1 through their HLH domain and inhibiting their transactivation potential [4], [10]. ID2 contributes to the input pathway of the circadian system as demonstrated by the abnormally rapid photoentrainment and increase in the magnitude of light-induced phase shifts exhibited by *Id2* null (*Id2−/−*) mice [4]. In addition, a role for ID2 in the output pathways of the circadian clock is suggested by altered expression profiles of CCGs in the liver of *Id2−/−* mice, including those involved in lipid metabolism [11]. Moreover, absence of *Id2* results in impaired adipogenesis *in vitro* [12] and reduced gonadal white adipose deposits and lipid content in liver in adult mice [11]. Due to these findings, the entwined nature of lipid and glucose metabolism and the interlocked relationship of the molecular circadian clock with metabolic processes [5], we hypothesized that ID2 contributes to the regulation of glucose homeostasis, energy utilization and the temporal regulation of feeding. It is well known that risk, development and manifestation of obesity, metabolic syndrome and insulin-resistance are sexually dimorphic, and animal models suggest contributing roles of both gonadal hormones and sex chromosomes [13]–[16]. Moreover, the effect of aging on the circadian clock and on glucose homeostasis has been reported in humans and animal models [17]–[20]. In the present study, our objective was to characterize key aspects of metabolic function and circadian regulation of activity and feeding in *Id2−/−* mice, and furthermore, to examine this in a sex- and age-specific context. We report here that *Id2−/−* mice have altered 24-hr patterns of locomotor and feeding behavior, and reduced weight gain despite elevated food intake, as well as sex-specific disturbances to adipocyte programing and to lipid accumulation in skeletal muscle and brown adipose tissue. We also report sex- and age-specific changes in glucose homeostasis, including fasting hypoglycemia, and increases in glucose tolerance, insulin sensitivity and glucose uptake that are most prominent in male animals.

## Results

### *Id2−/−* Mice Show Altered Daily and Circadian Patterns of Feeding and Locomotor Activity

**Figure 1:**
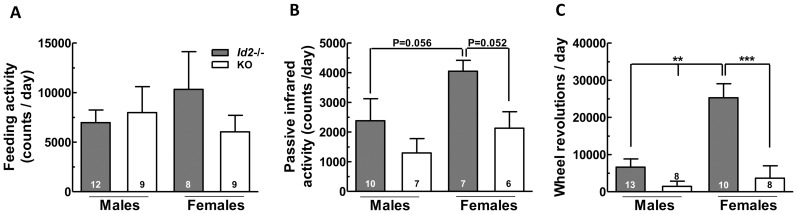
*Id2−/−* mice exhibit less locomotor activity compared to WT mice. A) Daily feeding activity counts of *Id2−/−* and WT mice (ANOVA: genotype, n.s.; sex, n.s.; interaction, n.s). B) Daily general activity counts in *Id2−/−* and WT mice determined by passive infrared motion detectors (genotype, P<0.05; sex, P = 0.057; interaction, n.s). C) Daily wheel revolution counts of WT and *Id2−/−* mice (genotype, P<0.001; sex, P<0.05; interaction, P<0.01). Values represent mean ± SEM. ***p<0.001.

Given that *Id2−/−* mice show altered circadian expression patterns in the liver of genes involved in metabolism [11], the daily [observed under light:dark (LD) cycle conditions] and circadian [observed under constant darkness (DD)] patterns of feeding and locomotor activity of *Id2−/−* mice were evaluated for possible alterations. When the mean values of the daily counts of feeding activity were analyzed, no significant difference was observed between genotypes (Fig. 1A). However, *Id2−/−* mice were found to be less active as demonstrated by the daily counts of general activity determined by passive infrared (PIR) motion detectors (Fig. 1B), and the wheel running activity (Fig. 1C), where *Id2−/−* mice exhibit significantly less activity compared to their wild type (WT) littermates. Interestingly, WT females showed higher general activity and wheel running activity compared to their male littermates (Fig. 1B, Fig. 1C). To account for these genotype- and sex-based differences in further analysis, the data for the daily and circadian activity patterns were normalized to percentages of average maximum activity.

**Figure 2:**
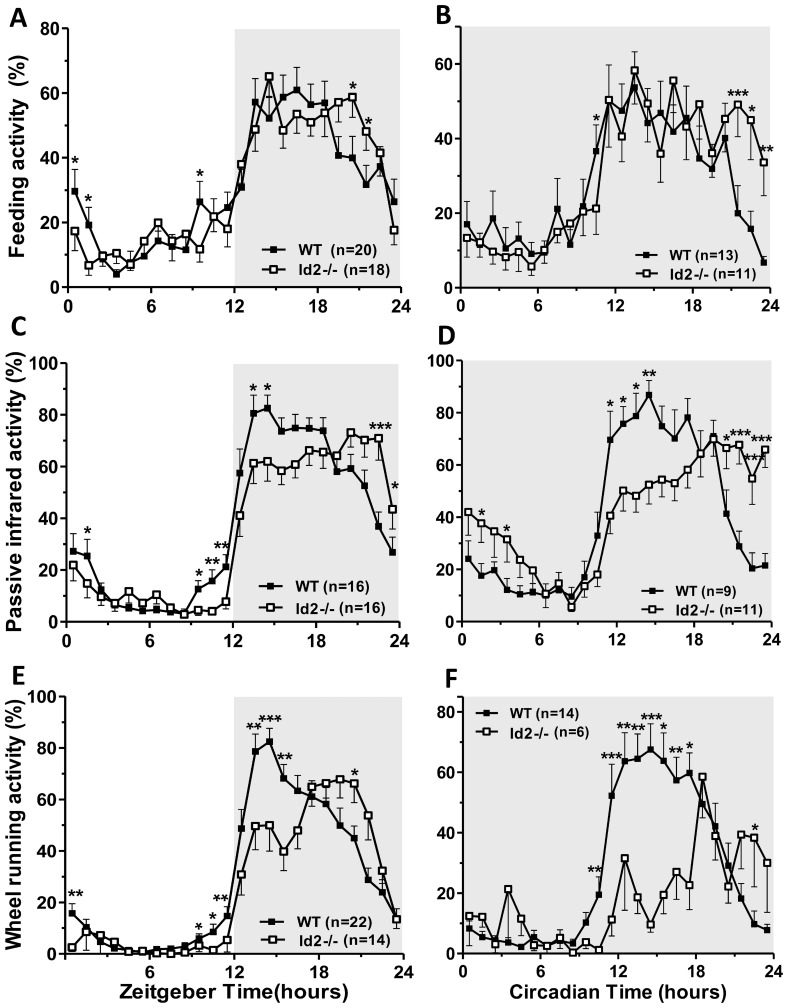
*Id2−/−* mice show altered daily and circadian patterns of feeding and locomotor activity. A) Daily feeding activity profile of WT and *Id2−/−* mice (ANOVA: time (T), P<0.001; genotype (G), n.s.; interaction (I), P<0.01). B) Circadian feeding activity profile of WT and *Id2−/−* mice (T, P<0.001; G, n.s.; I, P<0.05). C) Daily PIR motion detector general activity profile of WT and *Id2−/−* mice (T, P<0.001; G, P<0.05; I, P<0.001). D) Circadian general activity profile of WT and *Id2−/−* mice (T, P<0.001; G, P = 0.15; I, P<0.001). E) Daily wheel running activity profile of WT and *Id2−/−* mice (T, P<0.001; G, P<0.001; I, P<0.001). F) Circadian wheel running activity profile of WT and *Id2−/−* mice (T, P<0.001; G, P<0.01; I, P<0.001). The shaded area in the plots represents dark phase of the LD cycle or constant darkness. Values shown represent mean ± SEM. *p<0.05, **p<0.01 and ***p<0.001.

*Id2−/−* mice displayed extended daily feeding activity towards the late dark/night phase (ZT20 - ZT22) compared to their WT littermates (Fig. 2A). This pattern persisted in DD with the activity extending into the late subjective night (CT21 - CT0) (Fig. 2B). Consistent with the feeding activity patterns, the daily and circadian patterns of general movement were also altered in *Id2−/−* when compared to WT mice, where *Id2−/−* nocturnal activity persisted longer into the late dark phase (Fig. 2C, Fig. 2D). Even though *Id2−/−* mice displayed a profound reduction in wheel running activity, their daily and circadian wheel running profile extended towards the late dark phase (Fig. 2E, Fig. 2F). When the feeding and locomotor activity patterns under LD conditions were examined according to sex, we observed that *Id2*−/− females showed a more pronounced extended feeding and locomotor activity profile than *Id2*−/− males. The alterations among *Id2−/−* males and females were more consistent under DD conditions (Results S1, Fig. S1). Under LD conditions, we also observed a difference between genotypes in the level of activity during the light phase immediately prior to lights off (ZT12): a level of anticipatory activity was observed in the WTs 0–3 hrs before ZT12, a feature found reduced or absent in *Id2−/−* mice (Fig. 2, Fig. S1).

### Male *Id2−/−* Mice Show Less Body Mass Gain with Greater Food Intake

**Figure 3:**
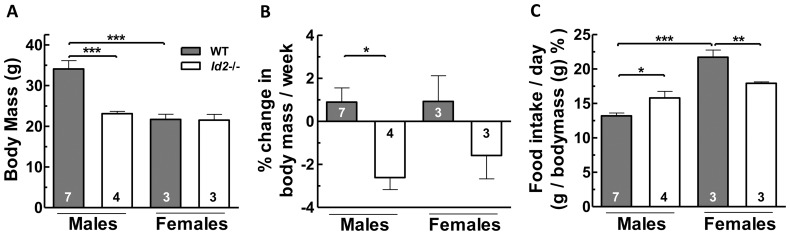
Resistance to weight gain in *Id2−/−* mice. A) Body weight comparison between WT and *Id2−/−* mice (ANOVA: genotype (G), P<0.05; sex (S), P<0.01; interaction (I), P<0.05). B) Weekly weight gain pattern of *Id2−/−* and WT mice in cages equipped with a running wheel (G, P<0.01; S, n.s.; I, n.s.). C) Daily food intake of *Id2−/−* and WT mice in wheel cages (ANOVA: G, P<0.01; S, n.s.; I, n.s). Values shown represent mean ± SEM. *p<0.05, **p<0.01 and ***p<0.001.

*Id2−/−* mice have been reported to show reduced body weight [4], [12]. In the current study, we observed this in a sex-specific manner between only male *Id2−/−* and WT mice (Fig. 3A). To assess whether there was any difference in the pattern of weight gain between WT and *Id2−/−* mice, we examined change in body mass in cages provided with a running wheel or in standard cages. In wheel cages *Id2−/−* males exhibited a significant weight loss compared to a gain observed in WT males. A similar pattern was observed between *Id2−/−* and WT females, although it was less pronounced and did not reach statistical significance (Fig. 3B). In standard cages, weight gain was not significantly different between genotypes and sexes (Fig. S2A). In wheel cages, *Id2−/−* males consumed more food and *Id2−/−* females consumed less food relative to body mass, when compared to WTs (Fig. 3C). The food intake in standard cages showed similar results, except that the difference between WT and *Id2*−/− females was not observed to be significant (Fig. S2B). We also examined the food intake during the light and dark phases of the LD cycle, and the differences in food consumption among genotypes and sexes were consistently observed in the dark phase (Results S1, Fig. S2C–F).

### Female *Id2*−/− Mice Show Differential White Adipocyte Size

**Figure 4:**
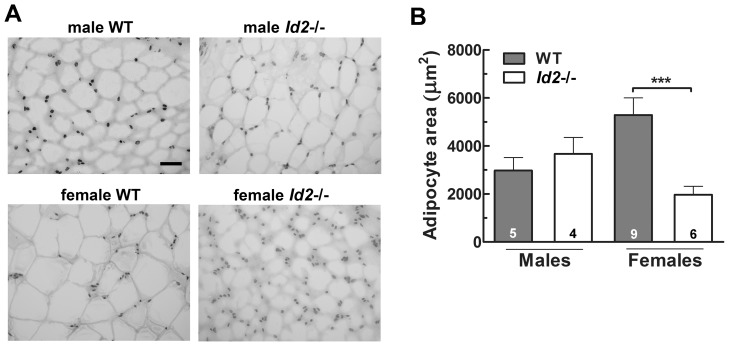
Cell size of the gonadal white adipose tissue is different between female *Id2−/−* and WT mice. A) Representative images of hematoxylin/eosin stained gonadal white adipose tissue sections of male and female WT and *Id2−/−* mice (scale bar = 50 µm). B) Cell area of gonadal WAT in WT and *Id2−/−* mice (ANOVA: genotype, P<0.05; sex, n.s.; interaction, P<0.001). No significant correlation was detected between cell size and age of animal (Spearman’s rank order correlation/linear regression, n.s.).

Our earlier report revealed that *Id2−/−* mice have less gonadal adipose deposits [11] (see also Fig. S6B). Histological evaluation of gonadal WAT revealed that the adipocyte size was comparable between genotypes for males, but that female *Id2*−/− cells were significantly smaller than female WT cells (Fig. 4).

### Glucose Tolerance and Insulin Sensitivity are Enhanced in Male *Id2−/−* Mice

**Figure 5:**
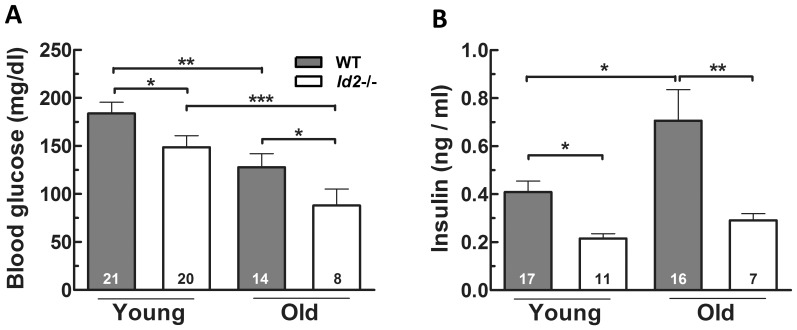
Fasting glucose and Insulin levels while aging. A) Fasting blood glucose levels of young and old, WT and *Id2−/−* mice (ANOVA: genotype, P<0.01; age, P<0.001; interaction, n.s.). B). Fasting insulin levels of young and old, WT and *Id2−/−* mice (genotype, P<0.001; age, P<0.05; interaction, n.s.). Values shown represent mean ± SEM. *p<0.05, **p<0.01 and ***p<0.001.

Following on from the observation of differences in the feeding pattern, food consumption and weight gain in *Id2−/−* mice, we wished to examine whether *Id2−/−* mice showed changes in glucose homeostasis. As age and sex are reported to influence insulin sensitivity [21], we compared glucose tolerance, insulin sensitivity and glucose-stimulated insulin secretion from both ‘young’ (2–5 months) and ‘old’ (10–22 months) groups of mice of both sexes. Our results revealed that the fasting blood glucose levels in the *Id2−/−* mice were significantly lower than age-matched WTs, and for both *Id2−/−* and WT mice, the fasting glucose levels decreased with age (Fig. 5A). Furthermore, fasting plasma insulin concentrations were found to be significantly higher in WT mice, which was further increased in older mice, whereas in *Id2−/−* mice fasting plasma insulin remained low even in older mice (Fig. 5B).

**Figure 6:**
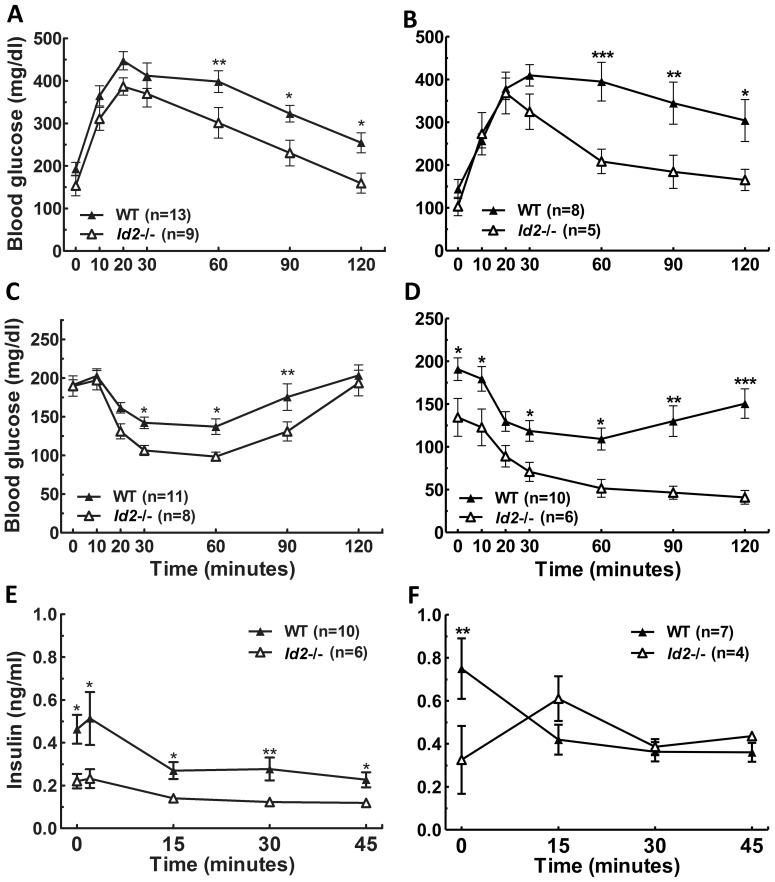
*Id2−/−* male mice display enhanced glucose tolerance and insulin sensitivity. A) Glucose tolerance test (GTT) of young male WT and *Id2−/−* mice (RM-ANOVA: time (T), P<0.001; Genotype (G), P<0.05; interaction (I), n.s). B) GTT of old male WT and *Id2−/−* mice (T, P<0.001; G, P<0.05; I, P = 0.001). C) Insulin tolerance test (ITT) of young male WT and *Id2−/−* mice (T, P<0.001; G, P<0.05; I, n.s.). D) ITT of old male WT and *Id2−/−* mice (T, P<0.001; G, P<0.01; I, P<0.001). E) Glucose-stimulated insulin release in young male WT and *Id2−/−* mice (T, P<0.01; G, P<0.01; I, n.s.). F) Glucose-stimulated insulin release in old male WT and *Id2−/−* mice (T, P = 0.103; G, P<0.01; I, n.s.). No effect of aging was observed in the glucose tolerance of either WT or *Id2*−/− males (RM-ANOVAs, n.s.). Comparison on young and old *Id2−/−* males reveal an increase in insulin sensitivity (T, P<0.001; A, P<0.001; I, P<0.01) in the older group. This large age effect was not observed in WTs (T, P<0.001; age (A), p = 0.06; I, P<0.05), although there was tendency for a slower recovery to baseline glucose levels at 90 and 120 mins (p<0.05). Values shown represent mean ± SEM. *p<0.05, **p<0.01 and ***p<0.001.

In both young and old mice a profound difference was observed between genotypes in responses to i.p. glucose tolerance testing (Fig. 6A, B). Male *Id2−/−* mice showed greater glucose tolerance when compared to age-matched WT males. In an i.p. insulin sensitivity test, young *Id2−/−* male mice displayed an enhancement in insulin sensitivity compared to WT young males, which was more pronounced in older *Id2−/−* males (Fig. 6C and 6D) and sometimes resulted in hypoglycemic-induced catatonic responses. Since we had observed a difference in the glucose tolerance and insulin sensitivity, we further examined plasma insulin concentrations after i.p. glucose injection in fasted male mice. Our data revealed reduced insulin release in young *Id2−/−* males in fasting insulin values and at all times after glucose injection, including at 2 min, when compared to WTs (Fig. 6E). Older *Id2*−/− males also showed a difference in the fasting insulin values when compared to WT males (Fig. 6F), but following glucose injection, maintained levels comparable between genotypes. Furthermore, in a separate set of measurements we found that the 2 min insulin values of older *Id2−/−* males were not significantly different from older WT males (*t*_(10)_ = 1.6, P = 0.148), although the mean value for WT males was 3.1-fold higher than *Id2−/−* males (Fig. S3A).

**Figure 7:**
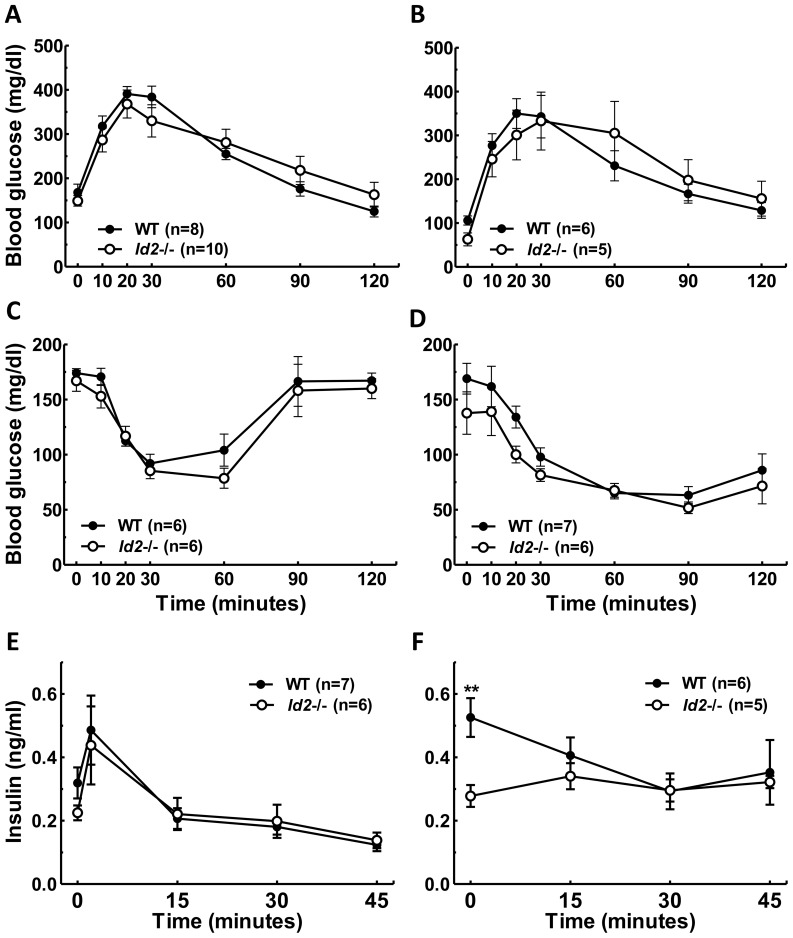
Glucose tolerance, insulin sensitivity and insulin release in *Id2−/−* females is unaltered. A) GTT of young female *Id2−/−* and WT mice (RM-ANOVA: time (T), P<0.001; genotype (G), n.s.; interaction (I), n.s.). B) GTT of old female *Id2−/−* and WT mice (T, P<0.001; G, n.s.; I, n.s.). C) ITT of young female *Id2−/−* and WT mice (T, P<0.001; genotype, n.s.; I, n.s.). D) ITT of old female *Id2−/−* and WT mice (T, P<0.001; G, P<0.01; I, n.s.). E) Glucose-stimulated insulin release in young female *Id2−/−* and WT mice (T, P<0.001; G, n.s.; I, n.s.). F) Glucose-stimulated insulin release in old female *Id2−/−* and WT mice (T, n.s.; G, P = 0.055; I, n.s.). No effect of aging was observed in the glucose tolerance of either WT or *Id2*−/− females (RM-ANOVAs, n.s.). An aging effect of insulin sensitivity was observed for WT and *Id2*−/− females (T, P<0.001; age, P<0.001; I P<0.001). Values shown represent mean ± SEM. **p<0.01.

No significant difference in glucose tolerance was observed between *Id2−/−* and WT female mice either in the young (Fig. 7A) or old groups (Fig. 7B). No significant effect of aging was observed in the glucose tolerance of either WT or *Id2*−/− females. Also, the response to an i.p. insulin sensitivity test was not different between genotypes in young females (Fig. 7C). Older *Id2−/−* females did not exhibit an enhancement in insulin sensitivity with respect to the overall response when compared to WTs (Fig. 7D). We observed a significant enhancing effect of age on insulin sensitivity that was comparable between WT and *Id2−/−* females (Fig. 7C,D). Consistent with the insulin sensitivity and glucose tolerance tests, glucose-stimulated insulin secretion in young *Id2−/−* females was also not found to be different from their WT littermates (Fig. 7E). Older *Id2−/−* females did not show any significant difference in their glucose-stimulated insulin secretion although they exhibited reduced basal insulin levels compared to older WT females (Fig. 7F). Also, in a separate set of experiments we found that the 2 min insulin levels of old *Id2−/−* females were not significantly different from WT females (*t*_(11)_ = 1.5, P = 0.167); however, the mean level of WT females was 1.6-fold higher than *Id2−/−* females (Fig. S3B).

### Glucose Uptake is Elevated in the Skeletal Muscle and Interscapular Brown Adipose Tissue of Male *Id2−/−* Mice

**Figure 8:**
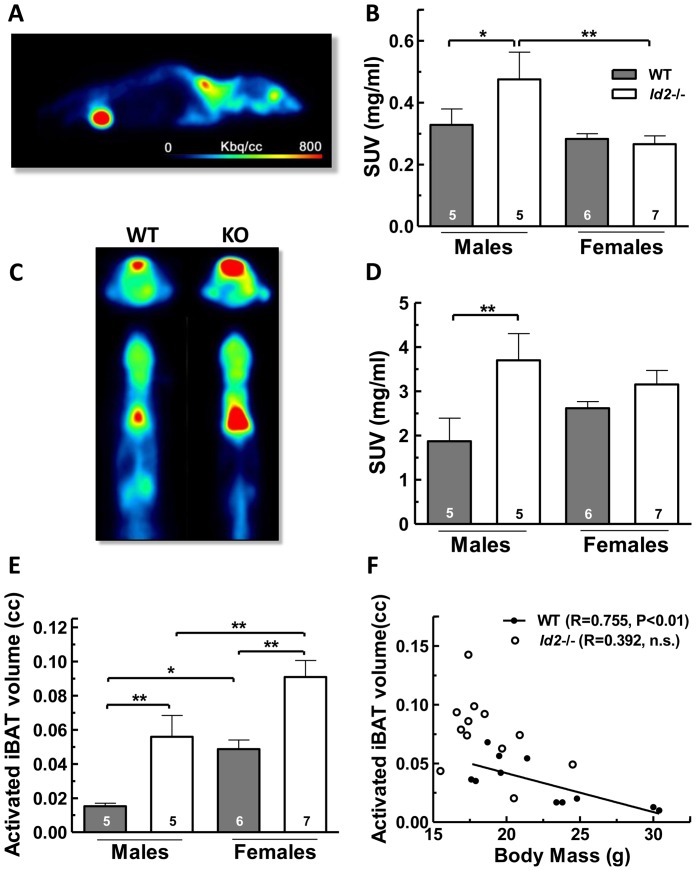
*Id2−/−* mice display elevated FDG uptake and activated volume of interscapular brown adipose tissue (iBAT). A) Shows a representative PET acquired image of FDG uptake in sagittal plane highlighting iBAT (dorsal) with high uptake. The injection site (right eye) and bladder are also visible. B) Quantitative analysis of FDG uptake in forelimb skeletal muscle in WT and *Id2−/−* mice represented as SUV (ANOVA: genotype (G), P = 0.152; sex (S), P<0.05; interaction (I), P = 0.108). C) Micro PET images of FDG uptake in iBAT at transverse (top) and coronal (bottom) planes for WT and mice. D) Quantitative analysis of the iBAT FDG uptake in WT and *Id2−/−* mice represented as standard uptake value (SUV) (G, P<0.01; S, n.s.; I, n.s.). E) Activated iBAT volumes obtained from micro PET studies of WT and *Id2−/−* mice (G, P<0.001; S, P<0.001; I, n.s.). F) Activated iBAT volumes of WT mice show a negative correlation with the body mass, which was not observed in the *Id2−/−* mice. Values shown represent mean ± SEM. *p<0.05 and **p<0.01.

Since *Id2−/−* mice showed greater insulin sensitivity, we evaluated glucose uptake in different tissues with known insulin responsiveness using MicroPET imaging (Fig. 8A). Quantification of FDG uptake (as standard uptake value [SUV] and determined from mean voxel values) by brain and heart revealed no significant difference in their respective SUVs between genotypes or sexes (Fig. S4A, S4B). However, FDG uptake by forelimb skeletal muscle was higher in *Id2−/−* males when compared to WT males, and was especially higher than that of *Id2−/−* females (Fig. 8B). When represented as SUV from maximum voxel value, this difference in FDG uptake by the skeletal muscle of *Id2−/−* males was found to be more pronounced (Fig. S4C). Interestingly, the SUV of WT skeletal muscle displayed a positive correlation with body mass and age (Spearman’s rank order correlation: R = 0.682, p<0.05; R = 0.712, p<0.05; n = 11). A remarkable enhancement of iBAT activity was observed in *Id2−/−* mice (Fig. 8C). Quantitation of FDG uptake in iBAT revealed a higher SUV for *Id2−/−* males compared to WT males (Fig. 8D). To eliminate the possible effect of a generally smaller body mass in *Id2−/−* males, we compared the FDG uptake between mass-matched male mice and found that the average SUV was 1.8-fold higher in *Id2−/−* males (body mass = 24.5g, SUV = 3.0 mg/ml) than WT males (body mass = 24.0±0.42g, SUV = 1.66±0.75 mg/ml, mean ± SEM). In addition, no correlation was observed between the SUV and the volume delineated for analysis, the body mass or age, either in WT mice or in *Id2−/−* mice, unless otherwise stated. Also, genotypic and sex differences in body mass were consistent with subjects used in the food intake/weight gain analysis (Fig. S4D).

The volume of activated iBAT was higher in *Id2−/−* mice of both sexes compared to WTs, indicating an elevated total FDG uptake by iBAT, irrespective of the mean SUVs (Fig. 8E). Furthermore, females showed greater activated iBAT volume than males in both genotypes (Fig. 8E). We also observed a negative correlation between body mass and activated iBAT volume in WT mice (Spearman’s rank order correlation: R = −0.745, P<0.01, n = 11) (Fig. 8F). This correlation was not observed in *Id2−/−* mice (R = −0.259, P = 0.402, n = 12), probably owing to their smaller body mass. No correlation was observed between the delineated activated volume and SUV of iBAT, either in WT mice or in *Id2−/−* mice, eliminating the possibility of any positive effect of high FDG uptake in the activated volume determination.

We evaluated the mass of iBAT from older mice and found that WT males had a greater iBAT weight (Fig. S5A). This suggested that the activated iBAT volume we have observed does not correspond to the amount of iBAT present, but the spread of its FDG uptake. The iBAT mass was found to increase with body mass in both WT and *Id2−/−* mice (Spearman’s rank order correlation: WT, R = 0.922, P<0.001, n = 15; *Id2−/−*, R = 0.898, P<0.001, n = 8). Consistent with the lean phenotype for *Id2−/−* mice, Oil Red O staining of iBAT from older mice revealed greater lipid deposition in WT males compared to *Id2−/−* males (Fig. S5B, S5C).

### Skeletal Muscle Triglyceride (TG) and Total Diacylglycerol (DAG) are Lower in Male *Id2−/−* Mice, and Proportions of Specific DAG Species are Altered

**Figure 9:**
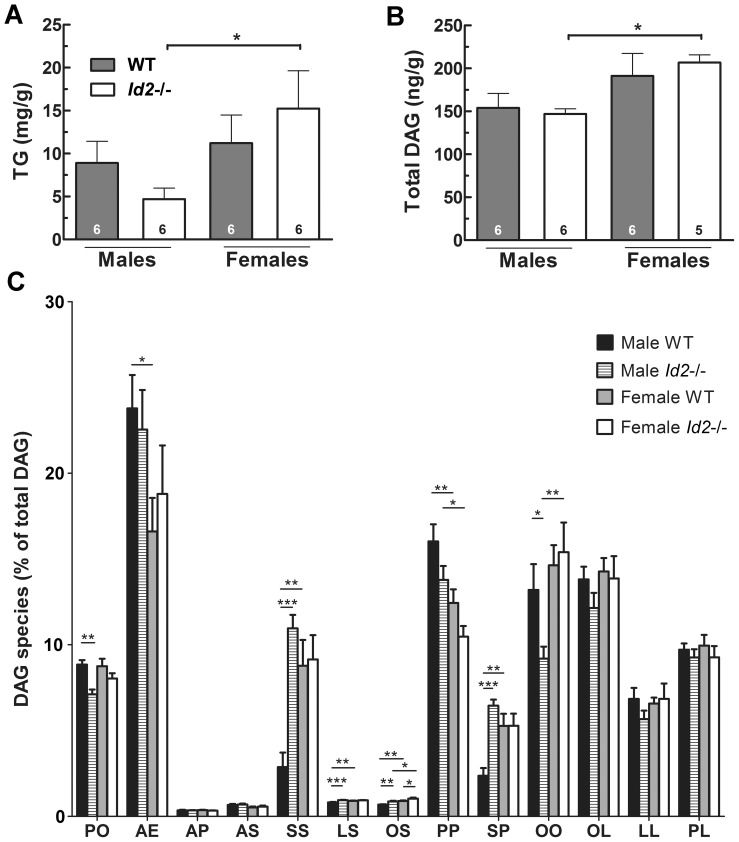
Skeletal muscle triglyceride (TG) and diacylglycerol (DAG) profiles in *Id2−/−* mice. A) Total TG content of tibialis anterior muscle (ANOVA: genotype (G), n.s.; sex (S), P<0.05; interaction (I), n.s.). B) Total DAG content of tibialis anterior muscle (G, n.s.; S, P<0.01; I, n.s.). C) DAG species analysis. DAG species are abbreviated as two contributing fatty acyl groups: A, E, S, O, L and P denote arachidonoyl, eicosapentanoyl, stearoyl, oleoyl, linoleoyl and palmitoyl groups, respectively. Values represent mean ± SEM. *p<0.05, **p<0.01 and ***p<0.001 are Turkey post-hoc tests following two way ANOVA for TG, DAG or each DAG species.

Intramyocellular DAG accumulation has been associated with insulin resistance, as DAG modulates intracellular insulin signaling and action, thereby decreasing insulin sensitivity [22]. We therefore analyzed the TG and DAG content of forelimb skeletal muscle to better understand the enhanced skeletal muscle glucose uptake and increased insulin sensitivity in the male *Id2−/−* mice. We observed a significant sex-specific difference between mice for both TG and total DAG content, and post hoc tests revealed a lower content in male *Id2−/−* mice as compared to female *Id2*−/− mice. (Fig. 9A, 9B). A detailed analysis of each specific DAG species revealed differences between sexes, but in particular between male *Id2*−/− and WT mice. For instance, in *Id2*−/− males DAG species containing the fatty acyl groups palmitoyl-oleoyl and oleoyl-oleoyl were reduced, and stearoyl-stearoyl, linoleoyl-stearoyl, oleoyl-stearoyl and stearoyl-palmitoyl were elevated (Fig. 9C).

## Discussion

ID2 is a HLH transcriptional repressor, rhythmically expressed in many mammalian tissues and previously reported to be involved in the input pathways, core clock function and output pathways of the circadian clock, and in adipogenesis [4], [10]–[12]. In the present study we have characterized an altered circadian rhythm and metabolic phenotype in *Id2−/−* mice, revealing a role for ID2 in the circadian regulation of feeding and locomotor behavior and in glucose metabolism.

Our studies show that *Id2−/−* mice exhibit lower levels of locomotor activity, and have extended activity patterns of feeding and locomotor activity. We also observed that WT females showed higher general activity and wheel running activity compared to WT males, a finding consistent with the reported sexual dimorphism in locomotor activity levels for various mouse strains, including C57BL/6J and 129x1/SvJ [23]. We also observed in *Id2−/−* mice a reduced level or absence of anticipatory activity 0–3 hr prior to the time lights off (ZT12). This finding is consistent with our earlier report in which *Id2−/−* mice were observed to have a significant ∼30 min delay in the phase angle of activity onset relative to WTs [4]. In WT mice the period of high activity is relatively short, peaking during the early dark/night phase of the LD cycle, in comparison with the longer and sustained profiles of *Id2−/−* mice, spanning early to late dark/night phase. That *Id2−/−* mice exhibit prolonged activity patterns of feeding and locomotor activity not only under LD but also DD conditions demonstrates the presence of an altered biological clock in *Id2* mutants.

There are several possible mechanisms that could explain the altered locomotor and feeding rhythms in *Id2−/−* mice. Given that ID2 plays a role in regulating circadian clock output in the liver, including metabolic CCGs, and because *Id2* is ubiquitously expressed and rhythmic [3], [4], [11], it is plausible that such a pivotal role is a feature of clocks throughout the body. This would include the hypothalamic brain and peripheral tissues involved in metabolic homeostasis.

Animals undergo vast changes in physiology to maintain metabolic homeostasis during the normal daily 24 hr feeding-fasting cycles and cycles of physical activity and arousal [5], [24]. Lack of precise time-of-day specific phase coordination between rhythmic processes within and between the metabolic organ systems (i.e. SCN, hypothalamic feeding-fasting centers, liver, WAT, BAT, skeletal muscle, pancreas, intestine), including impaired nutrient signaling (e.g. leptin, orexin, ghrelin), would predictably result in disruption of the normal balance of energy storage and utilization [5], [24]. Such disruption is thought to underlie the hyperphagic/obese phenotype of the *clock* mutant mouse [25]. It is noteworthy that ID2 can interact with CLOCK and its partner protein BMAL1 and modify both their localization signal and transactivation potential [10]. This general or perhaps tissue-specific disturbance in clock control may underlie both the behavioral and also physiological features (i.e. enhanced glucose uptake/reduced lipid storage) of the *Id2−/−* phenotype. We therefore suggest that the altered state of feeding and locomotor activity behaviors coupled with reduced physical activity, are a response to or the direct result of disruption in circadian metabolic homeostasis. These changes that we report in behavior would result in reduced energy expenditure and provide for a more sustained nutrient intake during the night. The most likely site for these changes in behavior is the SCN and hypothalamic feeding-fasting centers, known to regulate rhythmicity, wakefulness and feeding [5], [24]. Another possibility is that the behavioral changes reflect a disruption of normal SCN clock/output function [4], [10], [11], rather than a response to a system-wide metabolic alteration. Finally, reduced adipogenesis in the *Id2−/−* mouse [12] would likely compound any circadian disruption by reducing the lipid storage capacity of the animal. However, it is not sufficiently clear to what extent these processes can be considered distinct.

Our data revealed that *Id2−/−* males had a lower body weight while counter intuitively consuming greater relative quantities of food and simultaneously gaining less weight compared to WT males. This difference in body weight and weight gain was not observed in females. This observation could be explained in part by earlier findings of impaired adipogenesis and reduced adiposity in *Id2−/−* mice [11], [12]; and possibly by increased energy expenditure in BAT and skeletal muscle, as suggested by elevated FDG uptake specifically in *Id2−/−* males. This could also partly be attributed to the sex difference in the weight gain of WT mice, observed in C57Bl/6J mice, where males are more susceptible to obesity, and this advantage of females is abolished by ovariectomy [26]. Our results show that ablation of *Id2* reduces body mass gain in male mice. In fact there was a reduction in body mass in male *Id2*−/− mice in contrast to an increase observed in WT males. This weight loss was only detected in the presence of a running wheel, even though *Id2*−/− mice do not run in the wheel as much as WTs. The wheel could act as an enriched environmental stimulus to which the *Id2−/−* mice might show a greater response, since an enriched environment can improve metabolic health through increasing adaptive thermogenesis and browning of white adipocytes and thus increasing energy dissipation [27].

Earlier studies from our laboratory and others have shown that *Id2−/−* mice have less gonadal white adipose deposits [11], [12]. The present histological data show no significant difference in the gonadal adipocyte cell size between male WT and *Id2−/−* mice, suggesting that the reduced adiposity observed in male *Id2−/−* mice is due to a lower number of adipocytes. However, we did identify a sex-specific difference in which cells were smaller in female *Id2−/−* mice relative to female WT controls. Thus, reduced adiposity observed in female *Id2−/−* mice might be the result of a different mechanism, in which adipocyte number does not change but lipid accumulation does. Interestingly, the difference observed between female *Id2−/−* and WTs is consistent with reports of smaller adipocytes in *Id1*−/− and *Id4*−/− mice [28], [29]. However, in these studies either no clear indication of animal sex was provided or no differentiation of sexes reported. Clearly this underlies yet another sex-specific feature of the *Id2* null phenotype, that of differential adipocyte programing. These results could be attributed to the impaired adipocyte differentiation in the absence of *Id2*. It has also been observed that *Id2* promotes PPARγ expression, morphological differentiation and lipid accumulation during adipogenesis [12]. *Id2* gene expression is elevated in the initial phase of adipocyte differentiation and dramatically drops in the later phase [30], [31]. Here, ID2 potentially acts as a differentiation-inducing factor, as ID proteins are suggested to inhibit premature differentiation of progenitor cells and regulate cell fate determination [2]. A similar expression pattern has been observed for C/EBPβ, a key adipocyte differentiation inducer [32], which induces *Id2* expression during this process [33]. Interestingly, C/EBPβ null mice also exhibit reduced epididymal WAT deposits, impaired adipocyte differentiation and reduced lipid accumulation in BAT [34]. Moreover, specific clock genes contribute to adipogenesis, including BMAL1, which acts as negative regulator of adipogenesis [35], [36]. Since previous studies in our laboratory have indicated that ID2 can interact with BMAL1 and reduce its transactivation potential [4], [10], it raises the possibility that ID2 might positivity regulate adipogenesis via inhibition of BMAL1 activity.

Male *Id2−/−* mice displayed enhanced glucose tolerance, greater insulin sensitivity, low basal insulin levels and lower levels of glucose-stimulated insulin secretion when compared to WTs. Furthermore, glucose tolerance and insulin sensitivity were even more pronounced in aged male *Id2−/−* mice. Our results revealed that the fasting blood glucose levels in the *Id2−/−* mice were significantly lower than age-matched WTs, and for both *Id2−/−* and WT mice, the fasting glucose levels decreased with age. It is conceivable that this difference between young and old animals, irrespective of genotype, is a result of habituation to handling occurring in older animals [37]. However, there still remains a large reduction in fasting glucose in *Id2−/−* animals at each age-matched group. Furthermore, these collective glucose homeostasis tests, including basal and glucose-stimulated insulin levels, demonstrate that the ability of male *Id2*−/− mice to improve glucose tolerance is not associated with enhanced circulating insulin levels. In fact, surprisingly, in the young male *Id2*−/− mice, significantly *less* insulin is produced, thereby revealing a dramatically enhanced insulin sensitivity despite reduced insulin concentrations.

The enhanced glucose uptake by skeletal muscle and BAT of the male *Id2−/−* mice could contribute to the elevation in insulin sensitivity. Consistent with the weight gain pattern, female mice did not show any significant differences in the glucose homeostasis parameters when compared to WTs, apart from the lower basal insulin levels in the older group. Moreover the positive effect of aging on insulin sensitivity was observed in females irrespective of genotype. Our findings suggest the role of sex and age in the development of enhanced insulin sensitivity in the absence of ID2. This is consistent with the report that females are less susceptible to develop insulin resistance associated with diet-induced obesity, and where ovarian hormones are implicated [16].

An evaluation of glucose uptake by FDG-MicoPET imaging revealed higher FDG levels in the skeletal muscle of *Id2−/−* males. Considering the total mass of skeletal muscle in the body, a small difference in glucose uptake in the forelimb muscle could be indicative of a highly elevated glucose uptake by total skeletal muscle in *Id2−/−* males. Furthermore, insulin resistance in skeletal muscle is associated with aging [20], [38], [39], and sexual dimorphism is observed in the development of insulin resistance [40], [41]. Since *Id2* expression in the skeletal muscle is reported to increase during aging [42], it is possible that the difference between *Id2−/−* and WT males in skeletal muscle glucose uptake also increases with age. The fact that *Id2* is up-regulated in the skeletal muscle of obese type II diabetic mice further suggests a role of *Id2* in the development of skeletal muscle insulin resistance [43].

Accumulation of intramyocellular TG and especially DAG are associated with insulin resistance [22]. We found that the concentrations of these lipids were significantly reduced in the skeletal muscle of *Id2−/−* males as compared to *Id2−/−* females: 67% lower for TG, and 25% for total DAG. These data in part could explain both the enhanced insulin response and elevated skeletal muscle glucose uptake observed in the *Id2−/−* male but not female animals, through altered insulin signaling. Furthermore, the differences in the distribution of individual DAG species, observed between males and females (sex-specific differences), and especially between male *Id2*−/− versus male WT mice, reveal the complexity of the *Id2−/−* phenotype in terms of fatty acid composition. Interestingly, amongst several differences, the DAG species distribution data also indicate an elevated availability/metabolism of stearate (C18∶0) in the *Id2−/−* male compared to WT male controls. It has been suggested that alterations in specific DAG species may differentially contribute to insulin resistance [44], and so these disturbances in DAG distribution in the *Id2−/−* males could contribute to the increased glucose uptake by altering insulin signaling [11].

While the total DAG findings do not fully explain the genotypic differences we observed at the physiological level, they might in part constitute an explanation for the sex-specific element contributing to the glucose uptake and insulin sensitivity phenotype seen only in male *Id2−/−* mice. Likewise, these reductions in intramuscular TG/DAG may reflect reduced circulating lipids and/or increased myocyte mitochondrial fatty acid oxidation, as might be predicted from increased insulin sensitivity [44]. However, it still remains plausible that because of the intra-sex differences observed in DAG species composition between male *Id2−/−* and male WT mice, DAG might contribute to the genotypic differences. At the very least, low total DAG would be permissive to additional genotypic changes that are enhancing insulin sensitivity [42], [43].

We have observed that the average FDG uptake by iBAT is elevated in *Id2−/−* male mice. In addition, our data revealed a higher activated iBAT volume for *Id2−/−* mice of both sexes as well as the females of both genotypes. This is consistent with observations in adult humans, where females show increased mass of active iBAT [45]. However, since the actual mass of iBAT was lower in *Id2*−/− males and WT females than WT males, it is plausible that this elevated activated iBAT volume might also include recruitable brown adipocytes/beige adipocytes localized in the surrounding WAT mass [27], [46]. We also observed that the activated iBAT volume decreased with increase in body mass in WT mice, but not in *Id2−/−* mice. Similar observations were reported in humans, where an increase in body mass index (BMI) reduces the chance of detecting active BAT [45]; and BAT activity, measured as total FDG uptake, is negatively correlated with BMI and body fat percentage [47]. Our data from older mice revealed that WT male iBAT acquire greater weight and lipid deposits than *Id2*−/− males and WT females. We speculate that the presence of higher levels of lipid depositions in WT male iBAT might make it larger in size and less metabolically active per unit volume. In the *Id2*−/− male iBAT, higher glucose uptake, indicative of increased metabolic activity, suggests a higher thermogenic activity.

The situation in the *Id2*−/− male is indicative of the system-wide reduction in lipid accumulation, i.e. in addition to reduced quantities of WAT, this group has the lowest levels of skeletal muscle TG and DAG, and has low BAT lipids relative to WT males. Being that male *Id2*−/− mice on average have a smaller body mass, the changes in BAT physiology may be an adaptive response to an unfavorable surface area to mass ratio and compensating for increased loss of body heat [48]. It is plausible that to compensate for the increased energy expenditure, the mice increase food intake and reduce their locomotor activity. However, in the small cohort of body mass-matched male animals, male *Id2*−/− FDG uptake was still found to be elevated in respect to male WTs, suggesting that this tangible factor would not explain the entire extent of the BAT phenotype. Furthermore, in females, where there is no difference in body mass between mutant and WT animals, there still remains an elevation in activated BAT volume. In the *Id2*−/− females this increase in activated BAT volume also suggests a level increase in metabolic/thermogenic activity. Again, the observed reduction in activity might compensate for this increase energy expenditure.

Another mechanistic explanation for the observations in *Id2*−/− BAT and skeletal muscle and in glucose homeostasis would be associated with the circadian clock. As there are endogenous clock mechanisms within BAT, WAT and skeletal muscle [3], [8], [49], , the *Id2* null phenotype might be as a result of disturbances to their normal intrinsic temporal coordination of CCGs and subsequent metabolic process [49], [50]. As ≥7% of genes are rhythmically regulated in these tissues, including *Id2* [3], [49], [51], [52], and as *Id2* is implicated in regulating clock output [11], it is plausible that the absence of ID2 would result in a subset of CCGs being abnormally regulated, with potential consequences to local and systemic physiology.

Similar to the metabolic phenotype in *Id2−/−* mice, enhanced insulin sensitivity and increased glucose uptake by WAT and BAT were observed in a non-obese type 2 diabetic mice model in response to β3-adrenergic receptor activation [53]. Of note, β-adrenergic signaling induces *PGC-1α*, a thermogenic gene suggested to link the circadian clock and metabolism [54], and mice null for *PGC-1α* in WAT develop insulin resistance [55]. Interestingly, *PGC-1α* expression is elevated in *Id2−/−* liver [11].

In addition to *PGC-1α*, altered gene expression of other metabolic genes in *Id2−/−* liver could explain the present observations on *Id2−/−* mice. The rhythmically expressed genes *Igfbp1* and *Igfbp2*, encoding insulin like growth factor (IGF) binding proteins, are up-regulated in *Id2−/−* liver [11]; in the case of *Igfbp1,* a change in the peak phase of its rhythmic profile is observed. IGFBPs are predominantly inhibitory to IGF action, and IGFBP1 can inhibit IGF1-mediated differentiation of preadipocytes [56]. IGFBP2 can inhibit adipocyte differentiation *in vitro*, and when overexpressed *in vivo*, mice are protected from developing age- and diet-induced obesity and insulin resistance [57]. *Fibroblast growth factor* (*FGF*) *receptor 1* is also up-regulated in *Id2−/−* liver [11], interesting given that FGFs, including FGF21, are regulators of glucose and lipid metabolism [58].

In contrast to the metabolic phenotype observed in the absence of *Id2*, elevated expression of *Id2* is associated with obesity and/or diabetes. *Id2* expression in adipocytes is positively associated with obesity in mice and humans [12]. The *Id2* gene promoter contains a conserved CREB binding site, and its expression is stimulated in adipocytes by cAMP or a high fat diet [31], [59]. Also CREB is activated in adipocytes of obese mice where it promotes insulin resistance [59]. Increased *Id2* expression is observed in the muscle of obese type II diabetic mice and in the liver of type I diabetic mice [43], [60], and thiazolidinedione, a class of anti-diabetic drugs, inhibits *Id2* expression in human aortic smooth muscle cells [61]. Moreover glucose is reported to induce *Id2* expression in macrophages [62]. Taken together these findings clearly reveal the involvement of ID2 in the regulation of glucose/lipid metabolism and in the development of obesity and/or diabetes.

Similar to the *Id2−/−* mouse, a metabolic phenotype of reduced fat mass, increased insulin sensitivity and enhanced energy expenditure has been observed in *Id1* null mice. In contrast to ID2, ID1 is not required for adipocyte differentiation, and adipocyte differentiation is in fact accelerated in *Id1*−/− cells *in vitro* [29]. Defective adipogenesis is not implicated in the lower adiposity of *Id1*−/− mice [29]. Even though basal insulin levels are lower in *Id1*−/− mice, their glucose-stimulated insulin secretion is highly elevated, enhancing their glucose tolerance [63]. This is in contrast to the reduced levels of glucose-stimulated insulin we observed in *Id2−/−* mice. However enhanced gene expression of thermogenic proteins is also reported in *Id1*−/− mice [29], suggesting their BAT metabolic activity may be elevated as observed in *Id2−/−* mice. Similar to *Id2*, *Id4* is also reported to be necessary for adipogenesis, and *Id4*−/− mice exhibit reduced body weight and adipose deposits, including smaller adipocytes [28]. Loss of *Id3* does not affect weight gain or adipose deposit size in regular chow fed mice, but does result in reduced high fat diet induced visceral fat pad expansion [64]. Therefore the metabolic phenotypes observed in the absence of different ID proteins may not be the result of similar mechanisms, in spite of the possible functional redundancies amongst them.

Considering the involvement of ID2 in input pathways, core clock function and output pathway of circadian clock and also in adipogenesis [4], [10]–[12], we propose possible mechanisms for the development of the observed circadian and metabolic phenotypes in *Id2−/−* mice. Ablation of *Id2* could possibly alter the master clock in the SCN, thereby altering feeding and locomotor activity rhythms. Another possibility is an altered feeding pattern, either because of the altered CCG expression in periphery, as seen in liver [11], or centrally within the hypothalamic feeding centers [6], [24]; or because of a more persistent drive for feeding owing to lower energy storage capabilities (i.e. lipids), at least in part, due to reduced adipogenesis [48]. This would generate a drive for the animal to be active for a longer duration and thus alter locomotor activity rhythms. The lean phenotype in *Id2−/−* males could be a result of increased glucose uptake and utilization in both skeletal muscle and BAT, as well an increase in BAT thermogenesis. Likewise, there may be a generalized increase in metabolic activity, i.e. both glucose and fatty acid oxidation, as might be predicted from the increased insulin sensitivity and elevated FDG uptake. These changes in energy metabolism may reflect a disturbance in circadian clock function, occurring specifically within skeletal muscle and BAT [3], [8], [49], [50] and beyond these tissues, and thereby disrupting normal temporal coordination of metabolic processes; or the changes in energy metabolism may be a poor adaptation to the lower energy storage capabilities. The changes in glucose uptake coincide with enhanced insulin sensitivity and glucose tolerance in *Id2−/−* males. The sex-specific lower intramuscular total DAG, as well as differential profile of DAG species in male *Id2−/−* mice, might also contribute to the increased insulin sensitivity and glucose uptake; although a low concentration of intramuscular lipid in itself is a reflection of the lean state, most pronounced in the male *Id2−/−* mouse. The lean phenotype in *Id2−/−* males might also necessitate increased thermogenesis to compensate for a loss of body heat. It is plausible that to compensate for the increased energy expenditure, *Id2−/−* males increase food intake and reduce their locomotor activity.

In conclusion, we find that *Id2−/−* mice exhibit altered feeding and locomotor rhythms, fasting hypoglycemia, sex- and age-dependent enhanced glucose tolerance and insulin sensitivity, and sex-dependent elevated glucose uptake in skeletal muscle and BAT. Our observations clearly suggest the role of the ubiquitously expressed and rhythmic *Id2* gene in regulating pathways involved in glucose and lipid metabolism, as well as in the circadian control of feeding and locomotor activity behavior. BAT has received considerable attention recently as a potential therapeutic system to combat obesity, especially after it was discovered to occur in adult humans [65]. Our current data and earlier reports of *Id2* up-regulation during aging, suggests ID2 to be a potential therapeutic target for treating metabolic and circadian disorders in adults. Further, these data reinforce the relevance of sex and age-specific analyses in studying models of metabolic and circadian function as they pertain to human health and disease. In particular they also help to improve understanding of the development of obesity and diabetes, diseases prevalent in shift work personnel.

## Materials and Methods

### Ethics Statement

Animal experiments were approved by the University of Notre Dame Animal Care and Use Committee (protocol number 14–080) and performed in accordance with NIH Guidelines for the Care and Use of Laboratory Animals.

### Animals

The generation of *Id2* mutant mice and determination of genotypes were performed as described previously [4]. *Id2* WT and *Id2−/−* mice were on a mixed background for breeding purposes: 129sv/C57BL6J/FBVN [4] or 129sv/C57BL6J/FBVN/CD1 for skeletal muscle lipid analysis. The latter mice are lean, exhibit reduced gonadal WAT deposits, and males have a lower body mass (Figure S6), consistent with the previous analysis of *Id2*−/− mice [4], [11] and current study groups (Fig. 3, Fig. S4). Mice were provided with regular chow diet (Teklad Global diet 2919; kcal − 22% from fat, 23% from protein and 55% from carbohydrate) and sterile water containing antibiotic (sulfamethoxazole/trimethoprim oral suspension [Alpharma, Fort Lee, NJ] at a final dilution of 400 and 80 mg/liter, respectively) *ad libitum,* unless otherwise noted. The mice were entrained to a 12∶12 light: dark (LD) cycle with lights on at Zeitgeber time (ZT) 0 and lights off at ZT 12, and at a temperature of 20°–21°C and 50–65% humidity. In all experiments, *Id2*+/+ littermate animals were used as WT controls.

### Feeding and Locomotor Activity Recording

Mice (5–16 month old) were housed individually in 12∶12 LD cycle (light, 150–400 lux, fluorescent lights: General Electric 36-W cool white; dark, 0 lux) or DD (constant darkness, 0 lux) conditions. Feeding activity (visits to the hopper), passive infrared (PIR) motion detector general movement activity (*Slimline PIR motion detector*, Smarthome, Irvine, CA) and wheel running activity were simultaneously recorded every min for 20–30 days using specialized cages that were constructed in-house. Activity was monitored by a personal computer and using *Clocklab* hardware and software (Actimetrics, Wilmette, IL). Individual animal data of each minute from 5 consecutive days within 10–21 days into the experiment were selected for analysis of all three metrics. To account for the genotype and sex differences in the amplitude of activity (prominent in wheel running and PIR general activity), data from each individual were averaged into hourly bins and converted to percentage values, with the maximum values as 100% for each mouse. In DD conditions, the free running period length of each individual mouse was determined from wheel running activity or PIR activity, and data from this 5-day period were fitted into a 24-hr scale. The time of activity onset was determined by fitting lines to the actograms generated for 7–10 consecutive days in DD, either for wheel running activity or for PIR physical activity, and this was set to circadian time 12 (CT 12).

### Food Intake and Weight Gain Measurements

The mice (7–12 month old) were housed individually in cages with or without running wheels, with free access to water and regular chow. The day and night food consumption were determined by manual measurement of food every 12 hrs at ZT0 (lights on) and ZT12 (lights off) for at least 10 consecutive days. The mice were weighed every 3 days and the weekly weight gain was determined.

### Glucose and Insulin Tolerance Tests and Analysis of Insulin Release

For the glucose tolerance tests and the analysis of insulin release in response to glucose, mice were fasted 16 hrs prior to a baseline measurement of blood glucose or plasma insulin, eliminating the possible effect of difference in the time of food intake on the measurements. This was followed by intra-peritoneal injection of D-glucose (1.5 g/kg of body mass) and subsequent blood glucose measurements at 0, 10, 20, 30, 60, 90, and 120 min from a distal tail vein bleed. All glucose measurements were made with a handheld glucometer (One Touch Ultra, Milpitas, CA). Similarly for plasma insulin measurements, a series of blood samples were taken at 0, 2, 15, 30, and 45 min after the i.p. injection of glucose. The plasma separated from them was used for determining the insulin concentrations by ELISA (Crystal Chem, Inc., Downer’s Grove, IL). For insulin tolerance test the mice were fasted for 2 hrs and their baseline blood glucose levels were measured. This was followed by an i.p. injection of 0.75 units/kg body mass insulin (Humulin R, Eli Lilly and Co., Indianapolis, IN) and subsequent blood glucose measurements at the same time points as for the glucose tolerance test. All measurements were performed between ZT4 and ZT8. Mice were tested in two age cohorts, 2–5 and 10–22 months.

### MicroPET Studies

Positron emission tomographic (PET) and computed tomographic (CT) imaging was undertaken as described in Van der Veen et al, 2012 [9]. Mice (2–4.5 months) were fasted for 5 hrs prior to PET and CT scanning. After being anesthetized with 1.5% isoflurane, they were injected retro-oribitally with ∼200 µCi of (^18^F)-flurodeoxyglucose [FDG (Spectron MRC, South Bend, IN)] and returned to their cages. The mice were active within 2 mins after the light anesthesia. 30 mins after the injection, the mice were re-anesthetized and the PET and CT images were acquired on a trimodal Albira PET/SPECT/CT image station (Bruker Molecular Imaging, Billerica, MA). All procedures were performed between ZT6 and ZT10. High density PET (voxel size 0.65×0.65×0.944 mm [xyz]) and CT (voxel size 250 Hounsfield units) images were reconstructed and fused, volumes of interest (VOI) of relevant tissues were delineated and their FDG uptake was quantified using PMOD version 3.2 (PMOD Technologies, Zurich, Switzerland). The data of FDG uptake activity from the microPET images were expressed as standard uptake value (SUV) representing the average FDG uptake activity (mean voxel value) in each VOI (in kilo Becquerel per cubic centimeter) divided by injected dose of radioactivity (in Mega Becquerel) per kilogram of animal weight. For skeletal muscle we calculated the SUV from both the mean and maximum voxel values. The volumes of interest for brain and forelimb skeletal muscle were determined by manually contouring the tissue based on CT. For heart the VOIs were first delineated manually by contouring the FDG uptake activity that was clearly above the background activity followed by delineating a second VOI based on a threshold equal to 60% of the FDG uptake activity within the first, and the SUV of the second VOI is reported. Similarly the initial VOIs of the iBAT were delineated manually by keeping the heat bar to a fixed value to minimize inter-individual subjective bias, and the FDG uptake activity of this VOI is reported. To determine activated iBAT volume, a second VOI was selected based on a threshold equal to the average FDG uptake activity minus one standard deviation within the initial VOI, and the volume of this new VOI is used to report the activated iBAT volume [66].

### Histology

Gonadal WAT and iBAT were excised from older mice (10–26 months), embedded in OCT compound and frozen. Sections of 8 µm thickness were cut and stored at room temperature overnight for drying followed by fixation using chilled acetone method. The slides were then stained with Hematoxylin and Eosin. For lipid analysis the slides were stained in a working solution of Oil Red O (Sigma Aldrich) for 10 mins and then counterstained in Mayer’s Hematoxylin. Multiple images at 20×magnification were captured from each section using a Nikon 90i wide field microscope with a Nikon DS-Fi1 digital camera. Three to 7 sections from each individual were quantified. The images were analyzed manually using NIH ImageJ software. For WAT, the outline of each cell was drawn manually and area measured in pixels, and then converted to µm^2^ [67]. For iBAT lipid analysis the images were RGB stacked in ImageJ and the green channel was selected to measure the area covered by Oil Red O stain.

### Skeletal Muscle Lipid Analysis

Tibialis anterior were collected from mice (3–12 months) at ZT3-4 on liquid nitrogen and tissue samples analyzed for TG and DAG content by chloroform-methanol (containing butylated hydroxytoluene) extraction and liquid chromatography-mass spectrometry at the National Mouse Metabolic Phenotyping Center (MMPC) at Yale University School of Medicine (New Haven, CT).

### Statistics

Data were analyzed in Sigma Plot 12.0 software using two-factor ANOVA or two-factor repeated measures (RM) ANOVA with genotype, sex or age as the independent variables. The data that were non-normal and heteroscedastic were transformed to ranks (behavioral data), natural logarithms or square root values, before analysis. When ANOVA revealed a significant interaction between factors, Tukey’s *post hoc* tests were conducted. Student’s *t* test was used to compare two groups. The relationship between variables was analyzed using Spearman’s rank order correlation test or linear regression. The value of α was set at <0.05.

## Supporting Information

Figure S1 ***Id2−/−*** **males show more alterations in their circadian behavioral pattern than in their daily behavioral pattern**, **while** a**lterations in behavioral patterns of** ***Id2−/−*** **females are consistent under circadian and daily conditions.** A–E, males; F–K, females. A) Daily feeding activity profile of male WT and *Id2−/−* mice (ANOVA: time, P<0.001; genotype, P = 0.067; interaction, n.s.). B) Circadian feeding activity profile of male mice (time, P<0.001; genotype, P = 0.121; interaction, n.s.). C) Daily passive infrared (PIR) motion detector general activity profile of male mice (time, P<0.001; genotype, P<0.01; interaction, n.s.). D) Circadian general activity profile of male mice (time, P<0.001; genotype, P<0.01; interaction, P<0.001). E) Daily wheel running activity profile of male mice (time, P<0.001; genotype, P<0.001; interaction, P = 0.233). F) Daily feeding activity profile of female mice (ANOVA: time, P<0.001; genotype, n.s.; interaction, P<0.01). G) Circadian feeding activity profile of female mice (time, P<0.001; genotype, n.s.; interaction, n.s.). H) Daily general activity profile of female mice (time, P<0.001; genotype, n.s.; interaction, P<0.001). I) Circadian general activity profile of female mice (time, P<0.001; genotype, n.s.; interaction, P<0.01). J) Daily wheel running activity profile of female mice (time, P<0.001; genotype, P<0.001; interaction, P<0.001). K) Circadian wheel running activity profile of female mice (time, P<0.001; genotype, P<0.01; interaction, P<0.05). The shaded area in the plots represents dark phase of the LD cycle or constant darkness. Values shown represent mean ± SEM. *p<0.05, **p<0.01 and ***p<0.001.
(TIF)

Figure S2 **Weight gain and food consumption pattern of** ***Id2−/−*** **mice.** A) Weekly weight gain pattern of *Id2−/−* and WT mice in standard cages (ANOVA: genotype, n.s.; sex, n.s; interaction, n.s.). B) Daily food intake of *Id2−/−* and WT mice in standard cages (genotype, n.s.; sex, P = 0.076; interaction, P<0.05). C) Food consumption of *Id2−/−* and WT mice in standard cages during dark phase of LD cycle (genotype, n.s.; sex, n.s.; interaction, P<0.05). D) Food consumption of *Id2−/−* and WT mice in standard cages during light phase of LD cycle (genotype, n.s; sex, n.s.; interaction, n.s.). E) Food consumption of *Id2−/−* and WT mice in cages equipped with a running wheel, during dark phase of LD cycle (genotype, n.s; sex, P<0.001; interaction, P<0.01). F) Food consumption of *Id2−/−* and WT mice in wheel cages during light phase of LD cycle (genotype, n.s.; sex, P = 0.086; interaction, n.s.). Values shown represent mean ± SEM. *p<0.05, **p<0.01 and ***p<0.001.
(TIF)

Figure S3 **Insulin ELISA levels in aged animals at 2 min following glucose-treatment.** A) Insulin levels of old male WT and *Id2−/−* mice (t-test, n.s.). B) Insulin levels of old female WT and *Id2−/−* mice (n.s.). No significant effect of genotype or sex were detected for the insulin measurements when males and females were group analyzed (ANOVA: genotype, P<0.061; sex, P<0.075; interaction P<0.251), although there was a tendency for Id2−/− mice to have lower insulin levels. Values shown represent mean ± SEM.
(TIF)

Figure S4 **Quantitative analysis of FDG uptake.** A) FDG uptake in the brain of WT and *Id2−/−* mice (ANOVA: genotype, n.s.; sex, n.s.; interaction, n.s.). B) FDG uptake in the heart of WT and *Id2−/−* mice (genotype, n.s.; sex, n.s.; interaction, n.s.). C) FDG uptake in the forelimb skeletal muscle of WT and *Id2−/−* as SUV (from maximum voxel value) (genotype, P<0.01; sex, P<0.05; interaction, P = 0.074). D) Body weight comparison between WT and *Id2−/−* mice of mice used for FDG-PET analysis (ANOVA: genotype, P<0.05; sex, P<0.01; interaction, P<0.05). Values shown represent mean ± SEM. *p<0.05, **p<0.01 and ***p<0.001.
(TIF)

Figure S5 ***Id2−/−*** **males have reduced interscapular brown adipose tissue lipid deposits.** A) Comparison of iBAT weight of WT and *Id2−/−* mice (ANOVA: genotype, n.s.; sex, P = 0.03 P<0.05; interaction, P = 0.031 P<0.05). B) Representative images of Hematoxylin/eosin (H/E) stained and Oil Red O stained iBAT sections from male WT and *Id2−/−* mice (scale bar = 50 µm). C) Quantitative analysis of Oil Red O staining represented as percentage area fraction (genotype, n.s.; sex, P<0.05; interaction, P<0.01). C). Values shown represent mean ± SEM. *p<0.05, **p<0.01 and ***p<0.001.
(TIF)

Figure S6 **Body mass and gonadal WAT deposit mass of** ***Id2−/−*** **mice used for skeletal muscle lipid analysis.** A) Body weight comparison between WT and *Id2−/−* mice (ANOVA: genotype, P<0.001; sex, P<0.01; interaction, P<0.05). B) WAT mass of *Id2−/−* and WT mice as a proportion of body weight (ANOVA: genotype, P<0.01; sex, n.s.; interaction, n.s.). Values shown represent mean ± SEM. *p<0.05, **p<0.01 and ***p<0.001.
(TIF)

Results S1 (DOCX)
