# Supplementary figures and images for: Ablation of the Id2 Gene Results in Altered Circadian Feeding Behavior, and Sex-Specific Enhancement of Insulin Sensitivity and Elevated Glucose Uptake in Skeletal Muscle and Brown Adipose Tissue
Source: PLoS One. 2013 Sep 2;8(9):e73064. doi: 10.1371/journal.pone.0073064 (PMC3759459; doi:10.1371/journal.pone.0073064)

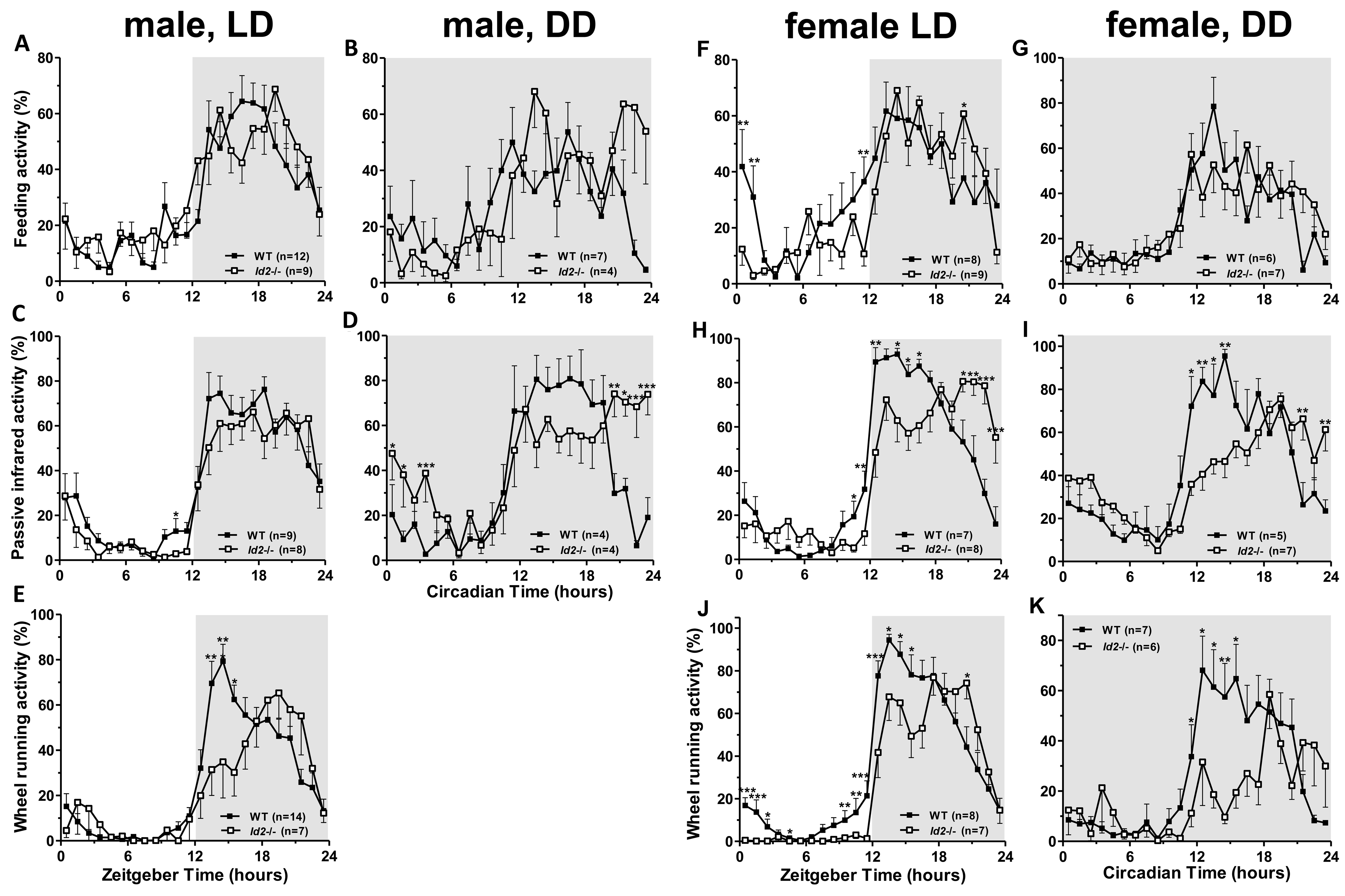

Supplement: Figure S1 — Id2−/− males show more alterations in their circadian behavioral pattern than in their daily behavioral pattern, while alterations in behavioral patterns of Id2−/− females are consistent under circadian and daily conditions. A–E, males; F–K, females. A) Daily feeding activity profile of male WT and Id2−/− mice (ANOVA: time, P<0.001; genotype, P = 0.067; interaction, n.s.). B) Circadian feeding activity profile of male mice (time, P<0.001; genotype, P = 0.121; interaction, n.s.). C) Daily passive infrared (PIR) motion detector general activity profile of male mice (time, P<0.001; genotype, P<0.01; interaction, n.s.). D) Circadian general activity profile of male mice (time, P<0.001; genotype, P<0.01; interaction, P<0.001). E) Daily wheel running activity profile of male mice (time, P<0.001; genotype, P<0.001; interaction, P = 0.233). F) Daily feeding activity profile of female mice (ANOVA: time, P<0.001; genotype, n.s.; interaction, P<0.01). G) Circadian feeding activity profile of female mice (time, P<0.001; genotype, n.s.; interaction, n.s.). H) Daily general activity profile of female mice (time, P<0.001; genotype, n.s.; interaction, P<0.001). I) Circadian general activity profile of female mice (time, P<0.001; genotype, n.s.; interaction, P<0.01). J) Daily wheel running activity profile of female mice (time, P<0.001; genotype, P<0.001; interaction, P<0.001). K) Circadian wheel running activity profile of female mice (time, P<0.001; genotype, P<0.01; interaction, P<0.05). The shaded area in the plots represents dark phase of the LD cycle or constant darkness. Values shown represent mean ± SEM. *p<0.05, **p<0.01 and ***p<0.001. (TIF) [file pone.0073064.s001.tif]

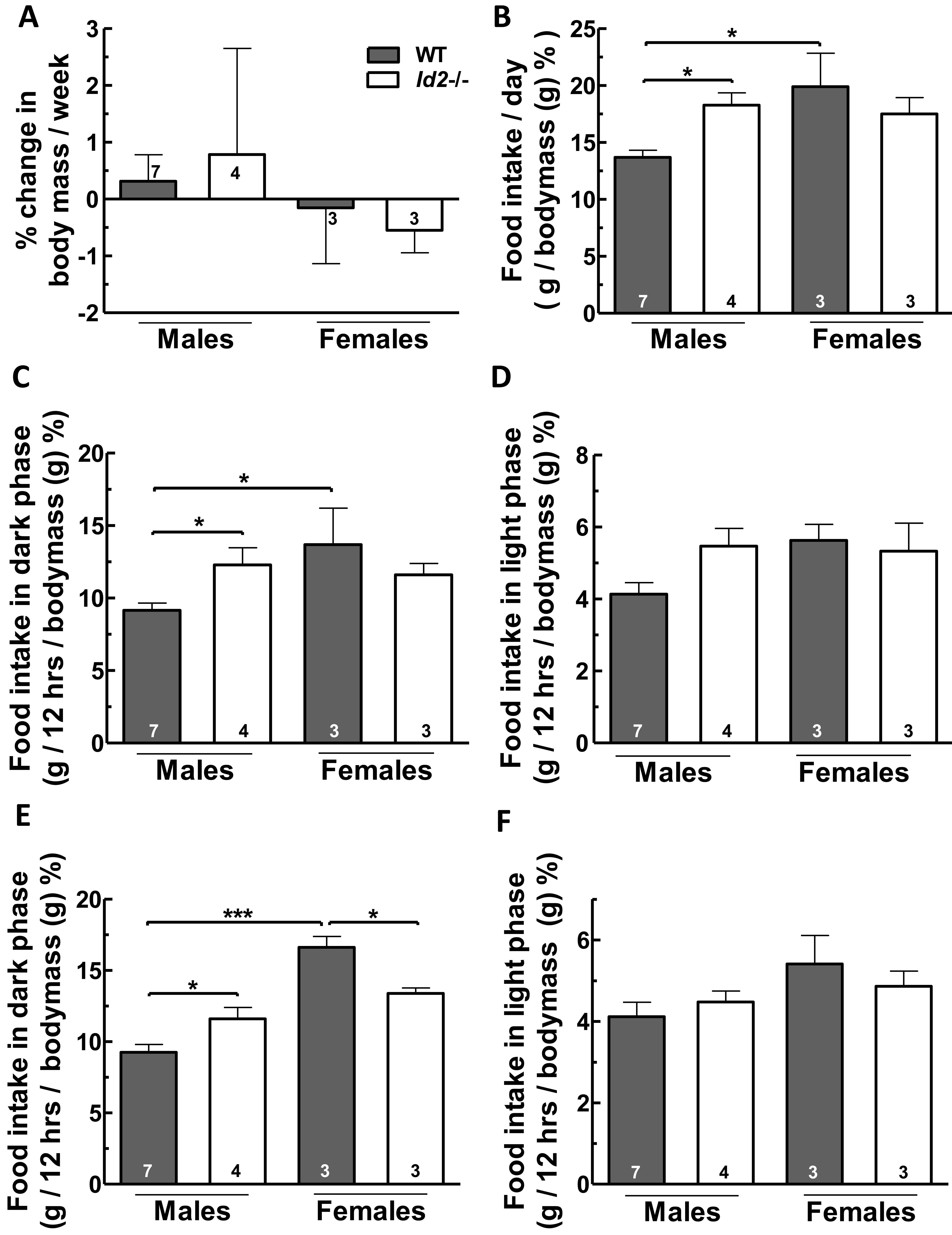

Supplement: Figure S2 — Weight gain and food consumption pattern of Id2−/− mice. A) Weekly weight gain pattern of Id2−/− and WT mice in standard cages (ANOVA: genotype, n.s.; sex, n.s; interaction, n.s.). B) Daily food intake of Id2−/− and WT mice in standard cages (genotype, n.s.; sex, P = 0.076; interaction, P<0.05). C) Food consumption of Id2−/− and WT mice in standard cages during dark phase of LD cycle (genotype, n.s.; sex, n.s.; interaction, P<0.05). D) Food consumption of Id2−/− and WT mice in standard cages during light phase of LD cycle (genotype, n.s; sex, n.s.; interaction, n.s.). E) Food consumption of Id2−/− and WT mice in cages equipped with a running wheel, during dark phase of LD cycle (genotype, n.s; sex, P<0.001; interaction, P<0.01). F) Food consumption of Id2−/− and WT mice in wheel cages during light phase of LD cycle (genotype, n.s.; sex, P = 0.086; interaction, n.s.). Values shown represent mean ± SEM. *p<0.05, **p<0.01 and ***p<0.001. (TIF) [file pone.0073064.s002.tif]

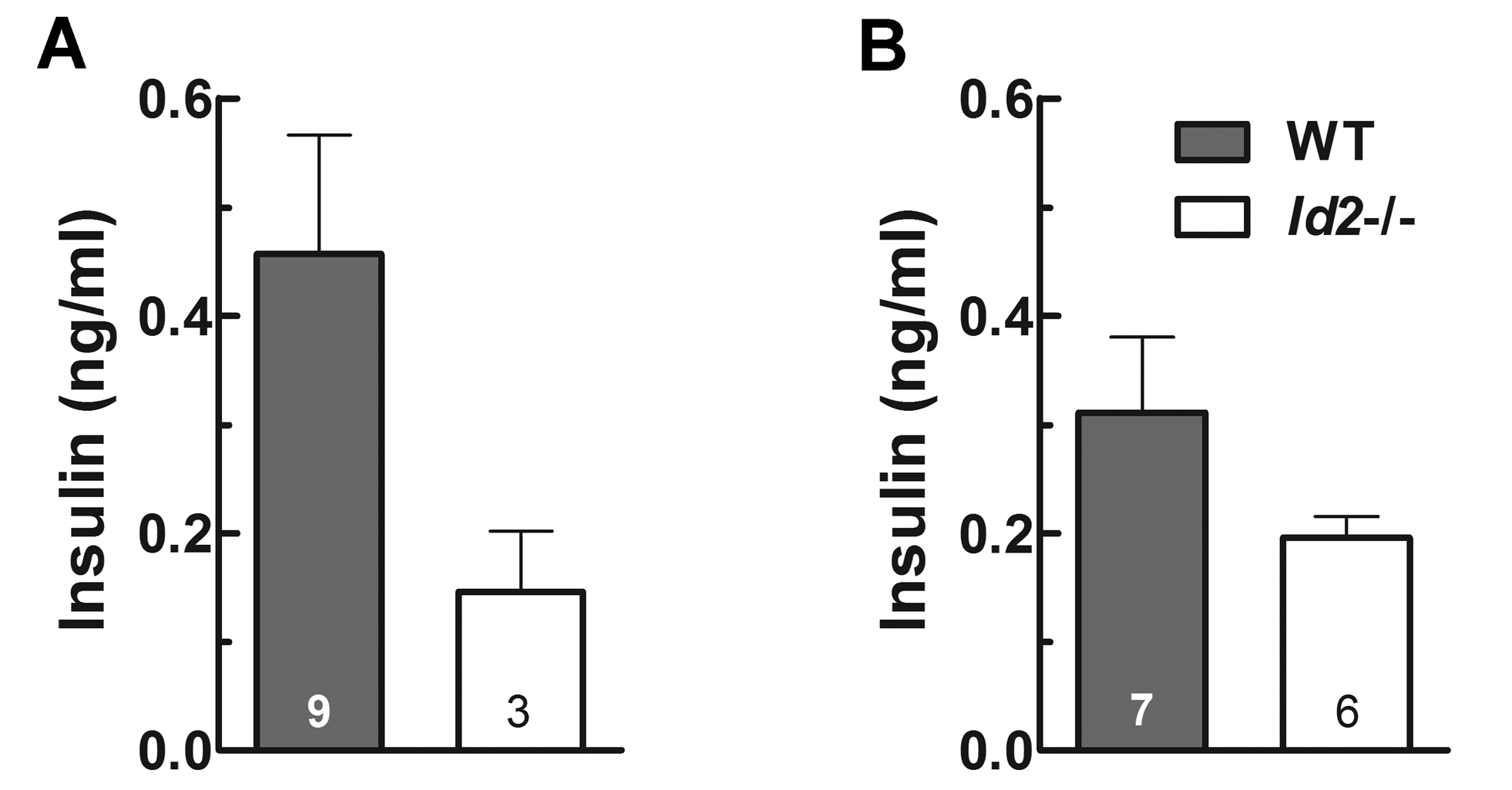

Supplement: Figure S3 — Insulin ELISA levels in aged animals at 2 min following glucose-treatment. A) Insulin levels of old male WT and Id2−/− mice (t-test, n.s.). B) Insulin levels of old female WT and Id2−/− mice (n.s.). No significant effect of genotype or sex were detected for the insulin measurements when males and females were group analyzed (ANOVA: genotype, P<0.061; sex, P<0.075; interaction P<0.251), although there was a tendency for Id2−/− mice to have lower insulin levels. Values shown represent mean ± SEM. (TIF) [file pone.0073064.s003.tif]

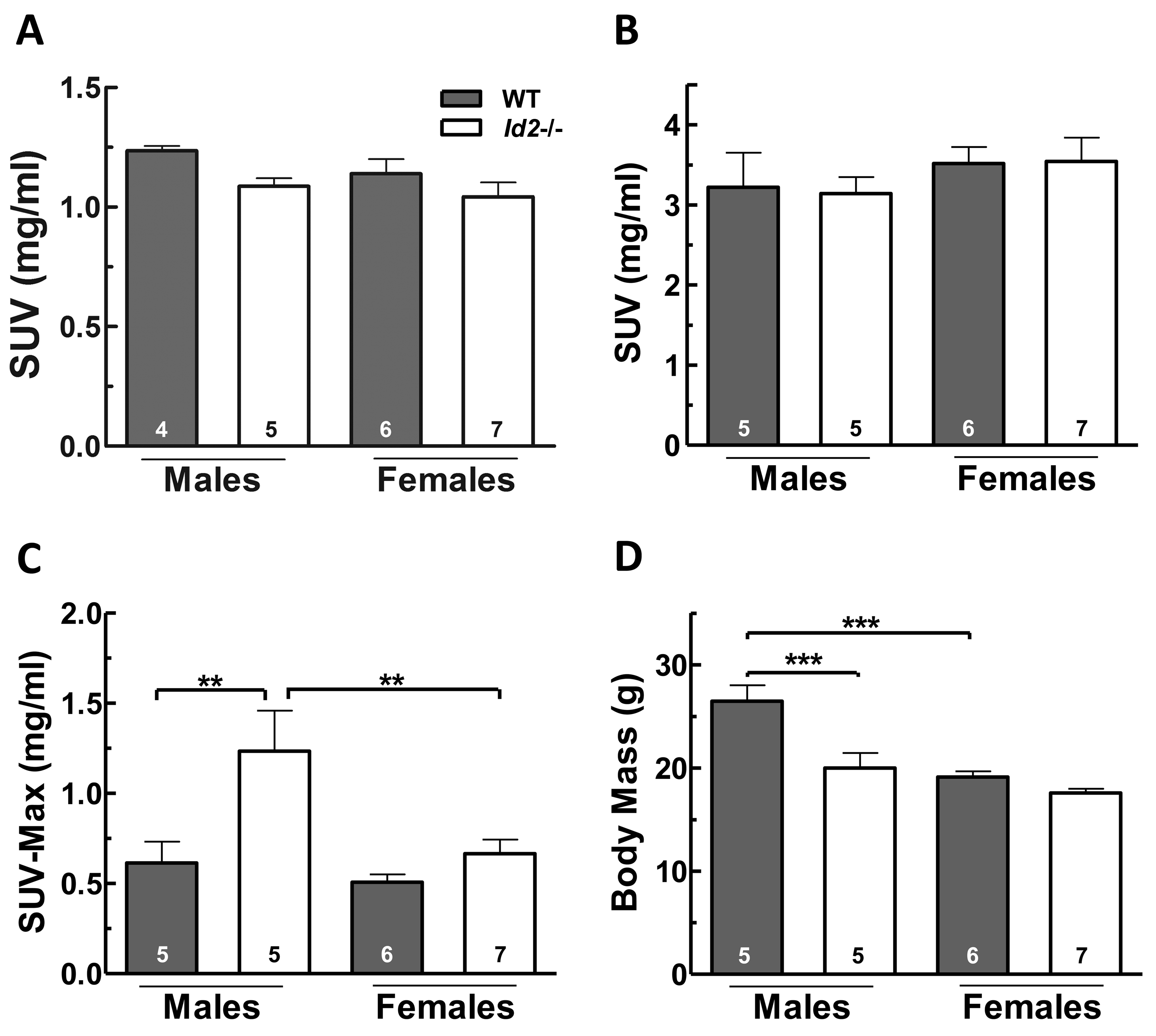

Supplement: Figure S4 — Quantitative analysis of FDG uptake. A) FDG uptake in the brain of WT and Id2−/− mice (ANOVA: genotype, n.s.; sex, n.s.; interaction, n.s.). B) FDG uptake in the heart of WT and Id2−/− mice (genotype, n.s.; sex, n.s.; interaction, n.s.). C) FDG uptake in the forelimb skeletal muscle of WT and Id2−/− as SUV (from maximum voxel value) (genotype, P<0.01; sex, P<0.05; interaction, P = 0.074). D) Body weight comparison between WT and Id2−/− mice of mice used for FDG-PET analysis (ANOVA: genotype, P<0.05; sex, P<0.01; interaction, P<0.05). Values shown represent mean ± SEM. *p<0.05, **p<0.01 and ***p<0.001. (TIF) [file pone.0073064.s004.tif]

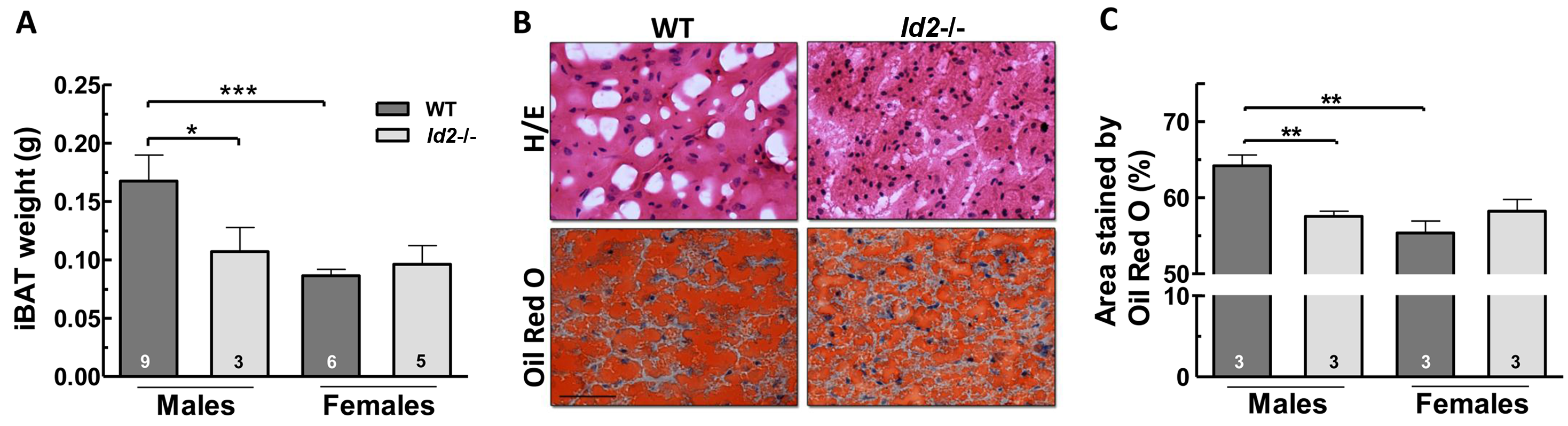

Supplement: Figure S5 — Id2−/− males have reduced interscapular brown adipose tissue lipid deposits. A) Comparison of iBAT weight of WT and Id2−/− mice (ANOVA: genotype, n.s.; sex, P = 0.03 P<0.05; interaction, P = 0.031 P<0.05). B) Representative images of Hematoxylin/eosin (H/E) stained and Oil Red O stained iBAT sections from male WT and Id2−/− mice (scale bar = 50 µm). C) Quantitative analysis of Oil Red O staining represented as percentage area fraction (genotype, n.s.; sex, P<0.05; interaction, P<0.01). C). Values shown represent mean ± SEM. *p<0.05, **p<0.01 and ***p<0.001. (TIF) [file pone.0073064.s005.tif]

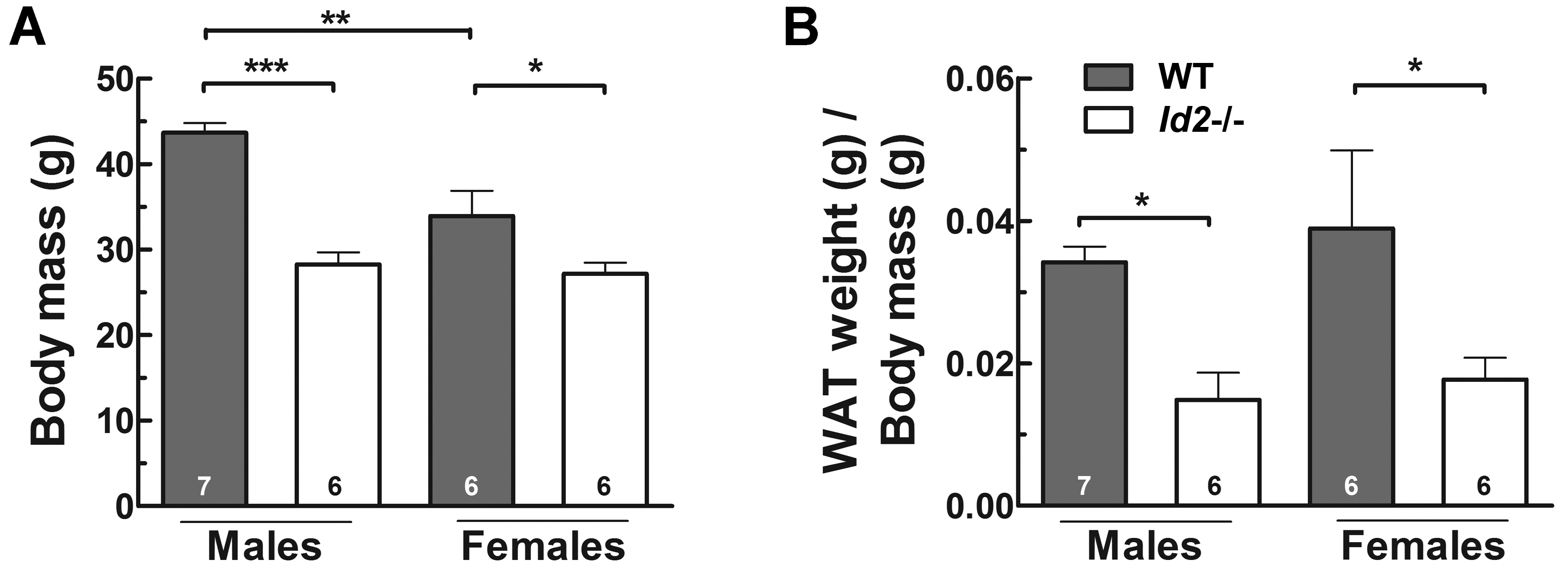

Supplement: Figure S6 — Body mass and gonadal WAT deposit mass of Id2−/− mice used for skeletal muscle lipid analysis. A) Body weight comparison between WT and Id2−/− mice (ANOVA: genotype, P<0.001; sex, P<0.01; interaction, P<0.05). B) WAT mass of Id2−/− and WT mice as a proportion of body weight (ANOVA: genotype, P<0.01; sex, n.s.; interaction, n.s.). Values shown represent mean ± SEM. *p<0.05, **p<0.01 and ***p<0.001. (TIF) [file pone.0073064.s006.tif]
