# Supplementary material for: Ablation of the Id2 Gene Results in Altered Circadian Feeding Behavior, and Sex-Specific Enhancement of Insulin Sensitivity and Elevated Glucose Uptake in Skeletal Muscle and Brown Adipose Tissue
Source: PLoS One. 2013 Sep 2;8(9):e73064. doi: 10.1371/journal.pone.0073064 (PMC3759459; doi:10.1371/journal.pone.0073064)
Supplement: Results S1 — (DOCX) [file pone.0073064.s007.docx]

**SI Results**

**Sex dependent difference in daily and circadian patterns of feeding and locomotor activity in *Id2*-/- mice.** Feeding and locomotor activity data were analyzed separately for both sexes to determine any sex-dependent differences. The *Id2*-/- males, when compared to WT males, did not show any significant difference in the daily feeding pattern (Fig. S1A). The circadian feeding patterns were also not observed to be significantly different, although *Id2*-/- male mice feeding profile tended to extend further into the late dark phase (Fig. S1B). Similarly, when PIR motion detectors were used under LD conditions, the general activity patterns of male *Id2*-/- mice showed an overall difference when compared to WTs. This included a delayed onset of activity and/or reduction of late daytime activity, but no significant extension of activity patterns was observed (Fig. S1C). In contrast the circadian profile of PIR general activity of *Id2*-/- mice showed profoundly extended pattern towards the late dark phase and the early light phase, when compared to WT males (Fig. S1D). We could not generate sufficient data for *Id2*-/- male circadian wheel running activity either because they tended not to run the wheel or they did not show any distinct ~24 hr rhythmic pattern. The data for the daily pattern of wheel running activity show an overall difference between WT males and *Id2*-/- males: even though the extended activity pattern was not observed to be significantly different from that of WTs, the balance between early versus late night activity was profoundly different (Fig. S1E).

In contrast to *Id2*-/- males, *Id2*-/- females exhibited significant extended feeding activity towards the late dark period under LD conditions (Fig. S1F). However, their circadian feeding activity patterns were not significantly different from WT females, even though they tended to feed in the late dark phase (Fig. S1G). The daily and circadian general activity patterns of *Id2*-/- females measured by PIR detectors were observed to be extended into the late dark phase when compared to WT females (Fig. S1H, I). Consistent with the feeding and PIR activity patterns, the wheel running activity of *Id2*-/- females were significantly extended towards the late dark phase (Fig. S1J). Furthermore, the balance between early versus late night activity was significantly different. Their circadian wheel running activity also showed this significant reduction in activity during the early night, as well as tended to extend towards the dark phase, although this was not observed to be significant in the multiple comparisons (Fig. S1K).

Under LD conditions the female *Id2*-/- mice exhibited more pronounced extended activity patterns than males. Similar to the difference observed between male *Id2*-/- and WT mice (Fig. S1C), female *Id2*-/- mice exhibited a reduction in late daytime anticipatory activity, observed in *both* PIR and wheel running activity profiles (Fig. S1H, J). The alterations in the feeding and locomotor activity patterns among *Id2*-/- males and females were more consistent under DD conditions.

**Food consumption in light and dark phases of the LD cycle.** We assessed the food consumption in animals provided with both standard caging and cages equipped with a running wheel under LD conditions. In standard cages during dark phase, WT males consumed less food relative to their body mass when compared to WT females and *Id2*-/- males (Fig. S2C), but the light phase feeding was not significantly different between genotypes or sexes (Fig. S2D). In the wheel running cages, the food intake in the dark phase by *Id2*-/- males was greater than the WT males. Also, WT females were observed to consume more food relative to body mass than *Id2*-/- females and WT males (Fig. S2E). The food intake in the light phase when housed in wheel cages was not significantly different between genotypes or sexes (Fig. S2F). In summary, the greater food intake by *Id2*-/- males exhibited both in standard and wheel cages were observed only in the dark phase.
